# Pathology explains various mechanisms of auto‐immune inflammatory peripheral neuropathies

**DOI:** 10.1111/bpa.13184

**Published:** 2023-06-25

**Authors:** Jean‐Michel Vallat, Stéphane Mathis

**Affiliations:** ^1^ Department and Laboratory of Neurology, National Reference Center for ‘Rare Peripheral Neuropathies’ University Hospital of Limoges (CHU Limoges) Limoges France; ^2^ Department of Neurology (Nerve‐Muscle Unit), ‘Grand Sud‐Ouest’ National Reference Center for Neuromuscular Disorders, ALS Center University Hospital of Bordeaux (CHU Bordeaux) Bordeaux France

**Keywords:** internode, myelin, neurofascin, node, nodoparanodopathy, paranode

## Abstract

Autoimmune neuropathies are a heterogeneous group of rare and disabling diseases in which the immune system is thought to target antigens in the peripheral nervous system: they usually respond to immune therapies. Guillain–Barré syndrome is divided into several subtypes including “acute inflammatory demyelinating polyradiculoneuropathy,” “acute motor axonal neuropathy,” “acute motor sensory neuropathy,” and other variants. Chronic forms such as chronic inflammatory demyelinating polyneuropathy (CIDP) and other subtypes and polyneuropathy associated with IgM monoclonal gammopathy; autoimmune nodopathies also belong to this group of auto‐immune neuropathies. It has been shown that immunoglobulin G from the serum of about 30% of CIDP patients immunolabels nodes of Ranvier or paranodes of myelinated axons. Whatever the cause of myelin damage of the peripheral nervous system, the initial attack on myelin by a dysimmune process may begin either at the internodal area or in the paranodal and nodal regions. The term “nodoparanodopathy” was first applied to some “axonal Guillain–Barré syndrome” subtypes, then extended to cases classified as CIDP bearing IgG4 antibodies against paranodal axoglial proteins. In these cases, paranodal dissection develops in the absence of macrophage‐induced demyelination. In contrast, the mechanisms of demyelination of other dysimmune neuropathies induced by macrophages are unexplained, as no antibodies have been identified in such cases. The main objective of this presentation is to show that the pathology illustrates, confirms, and may explain such mechanisms.

## Graphical abstract

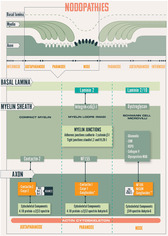

## 1 INTRODUCTION

Peripheral neuropathies represent a highly prevalent neurological problem in the general population (incidence: 77/100,000 inhabitants/year; prevalence: 1%–12% in all age groups and up to 30% in older people) [1], because of the high prevalence of some etiologies such as diabetes mellitus, alcoholism, neurotoxic substances (medications, chemotherapies), and renal insufficiency. Other rare causes of peripheral neuropathy may be hereditary (Charcot–Marie–Tooth disease, etc.) or acquired (immune‐mediated, neoplastic, infectious, etc.).

Immune‐mediated diseases may affect the peripheral nervous system by various mechanisms (inflammatory, vascular, etc.) being induced by a process involving cellular and humoral immunities. After a presentation of the main normal histological structure of the myelinated nerve fibers, we discuss here the various pathological aspects of some autoimmune neuropathies. The main objective of this presentation is to show that the pathology illustrates, confirms, and may explain various mechanisms concerning this type of neuropathy.

## 2 THE NORMAL MYELINATED PERIPHERAL NERVE

**FIGURE 1:**
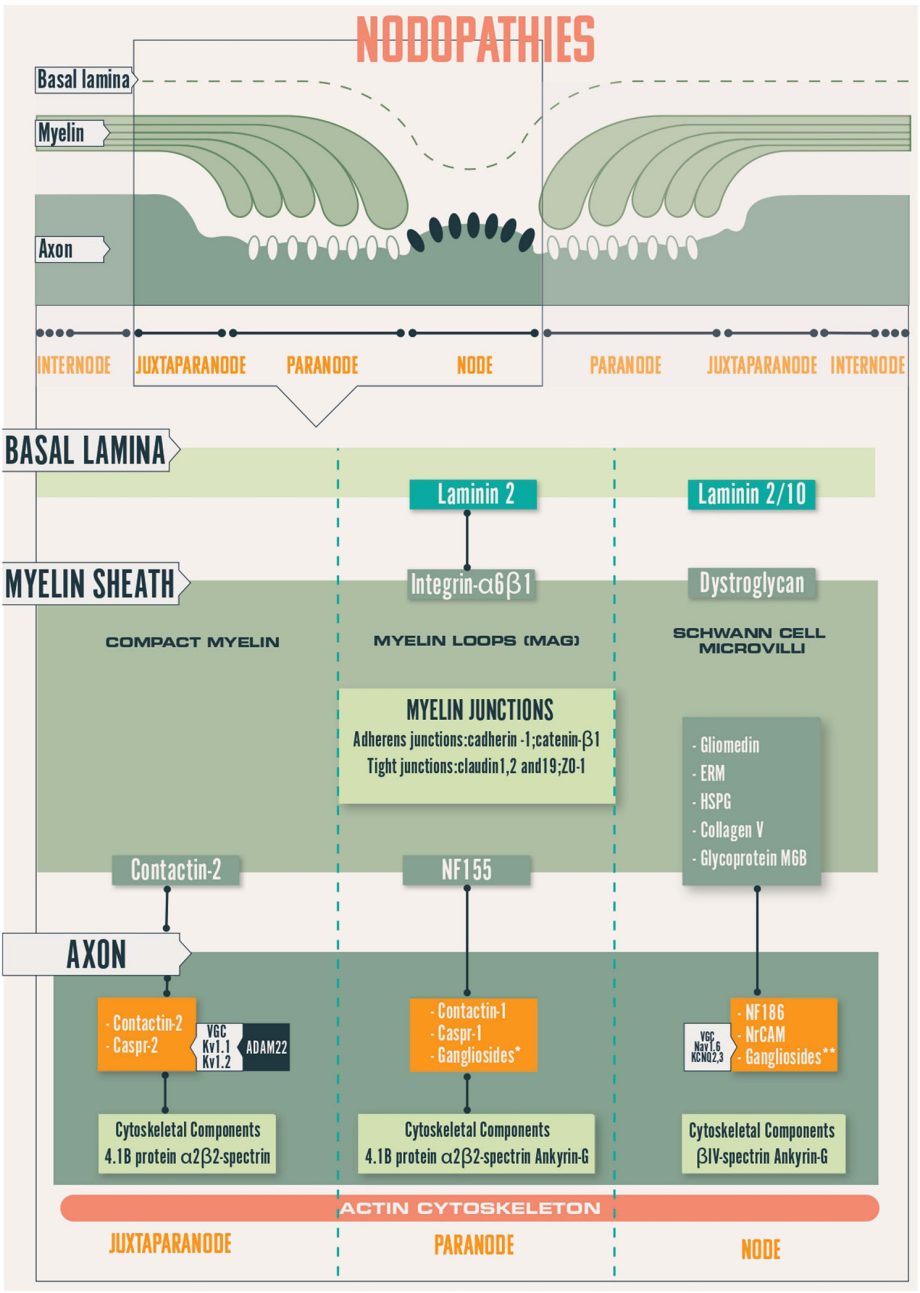
Schematic representation of the node of Ranvier. Caspr, contactin‐associated protein; ERM, Ezrin‐Radixin‐Moesin; KCNQ, potassium channel, voltage‐gated, KQT‐like subfamily Kv, potassium channel; MAG, myelin‐associated glycoprotein; Nav, sodium channel; NF155, neurofascin‐155; NF186, neurofascin‐186/140; VGC, voltage‐gated channel.

Myelinated axons are organized in distinct domains including nodes of Ranvier (NoR), paranodes, juxtaparanodes, internodes, characterized by specific molecular complexes (Figure 1). The internode represents 99% of the length of the myelinated Schwann cell (SC), the remaining 1% corresponding to the nodal region [2]. The myelin sheath is made up of regular concentric lamellae with a 12–17 nm periodicity; it is a modified cell membrane and each lamella arises from the spiral wrapping of the SC plasma membrane around the axon. Myelin provides reliable electrical insulation of the axons and thereby ensures rapid propagation of nerve impulses through the NoRs.

**FIGURE 2:**
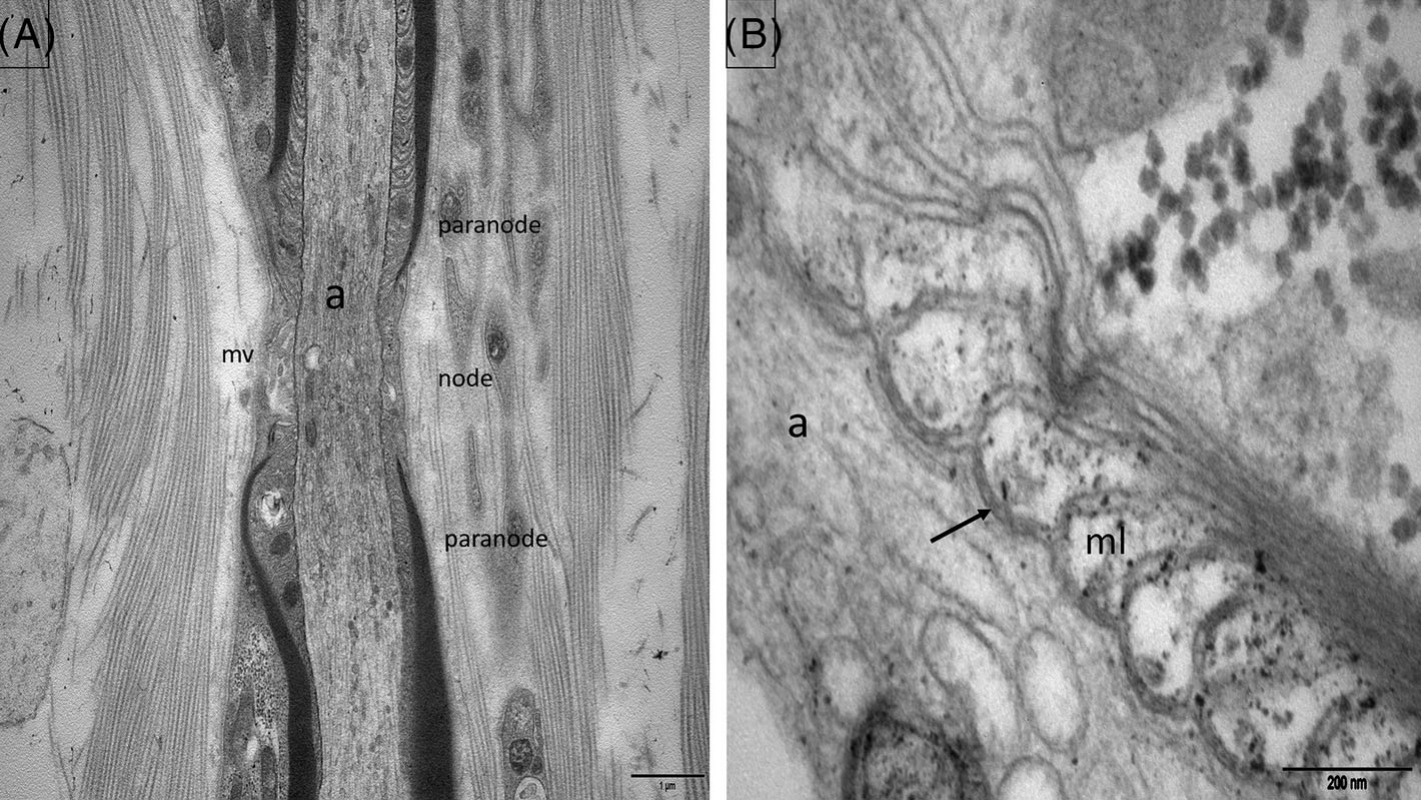
Normal control subject. Electron micrographs, longitudinal sections of the nodoparanodal area (sural nerve biopsy). (A) Paranode, node, microvilli (mv), axon (a). (B) Myelin (ml) loops are attached to the axolemma by periodic, punctiform, dense structures corresponding to the transverse bands (arrow).

The nodal region ensures by saltatory conduction, the long distance rapid transmission of impulses with the least expenditure of energy. At this level, the myelin sheath is interrupted so that the axon is in direct contact with the microvilli which are small, elongated finger‐like structures emanating from the SC cytoplasm. Between two NoRs, the area corresponding to a myelinated axon is called the “internode.” At the two ends of this internode are characteristic regions. First, the paranodes (where the myelin lamellae terminate by a series of pockets called the “myelin loops,” or “paranodal loops”) are attached to the axolemma with a gap of 2.5–3 nm (septate junctions) by small periodic, punctiform, and dense structures like dots, called the “terminal bands” (TB) (Figure 2). At this level, the axolemma presents numerous voltage‐gated sodium (Nav) channels. The paranodal junctions are composed of three major proteins: contactin‐1 (CNTN1) and contactin‐associated protein‐1 (Caspr1) on the axonal side and neurofascin 155 (NF155) on the myelin loops. A neurofascin neuronal 186 kDa variant (NF186) is located at the nodes; it helps to stabilize and maintain the nodal Na + channel protein complex. At the juxtaparanodes, voltage‐gated potassium (Kv) channels are anchored and clustered by contactin‐associated protein 2 and transient axonal glycoprotein 1 (Figure 1). Gangliosides GM1 and GD1A are located on nodal and paranodal axolemma, SC microvilli, and abaxonal myelin.

## 3 CLASSIFICATION OF THE PERIPHERAL NEUROPATHIES

Since the end of the 19th century, peripheral neuropathies have been classified according to their clinical characteristics, leading, over time, to the following possibilities: symmetrical (polyneuropathy) or asymmetrical (mononeuropathy and multiple mononeuropathy), involvement including nerve roots (polyradiculoneuropathy) and involvement primarily affecting the cell bodies (neuronopathy). Peripheral neuropathies can also be classified according to their mode of onset: acute (onset in less than 4 weeks), subacute (onset in 4 to 8 weeks), chronic (onset in more than 8 weeks), or relapsing–remitting. Since the middle of the 20th century, with the development of clinical neurophysiology, peripheral neuropathies are traditionally classified as “demyelinating” or “axonal,” according to whether the pathologic process affects primarily the myelin/SC or the axon [3].

In the 1990's, this traditional dichotomy (“axonal” vs. “demyelinating”) has been questioned when some subtypes of Guillain–Barré syndrome (GBS) were identified as “*axonal GBS*” (nowadays known as “acute motor axonal neuropathy” [AMAN] or “acute motor and sensory axonal neuropathy,” [AMSAN]) [4]. However, in AMAN/AMSAN, the primary attack is directed toward the excitable nodal axolemma, inducing a temporary failure of conduction at the NoR named “reversible conduction failure” (RCF), possibly because of loss of Nav channels, (distinguishing it from the classical demyelinating conduction block); secondary, there may be (in the more severe cases) axonal degeneration (giving an electrophysiological pattern of axonal neuropathy) [3]. The recent discovery of pathogenic antibodies targeting the nodal and/or the paranodal regions [5] has led to the recognition of a new group of antibody‐mediated neuropathies, nowadays called “nodoparanodopathies”: this term was first proposed to characterize a common pathogenic mechanism of dysfunction/disruption at the NoR resulting in a pathophysiological continuum from transitory nerve conduction failure to axonal degeneration [3]. It is difficult to determine the incidence of this new entity, which may be around 5% of GBS and chronic inflammatory demyelinating polyneuropathy (CIDP) patients. A new classification of autoimmune peripheral neuropathies based on the involved domains of the myelinated fiber and, when known, on the antigen has now been presented [6].

## 4 THE PATHOLOGICAL FEATURES OF INTERNODOPATHIES

### 4.1 The classical Guillain–Barré syndrome (GBS)

Guillain–Barré syndrome is an acute‐onset, monophasic immune‐mediated polyradiculoneuropathy clinically characterized by a rapidly progressive, bilateral weakness of the limbs and generalized hyporeflexia or areflexia, progressing over several days to 4 weeks. The “classical GBS” is known as “acute inflammatory demyelinating polyradiculoneuropathy” (AIDP), first fully described in 1916 by Guillain, Barré, and Strohl [7, 8]. In the 1950's, Asbury et al. were the first to support the idea that GBS may be a “cell‐mediated immunologic disorder, in which peripheral nervous tissue, in particular myelin, is attacked by specifically sensitized lymphocytes” [9]. In most cases of GBS, nerve biopsy (NB) is not done, because it is usually a readily recognizable syndrome on clinical and electrophysiological criteria: NB may be of interest in atypical cases, especially asymmetrical ones where a vasculitic process may be suspected as a differential diagnosis. Pathologically, AIDP is characterized by inflammatory infiltrates and segmental demyelination (with a variable amount of axonal damage, depending upon the intensity and destructiveness of lesions) [10, 11, 12]. Involvement of the PNS can extend from the spinal root to intramuscular nerve endings and may result in damage to distal sensory axons and spinal and sympathetic ganglia.

In AIDP, the primary site of immune attack (cellular and/or humoral) is the SC surface. There are a few endoneurial mononuclear cell infiltrates composed of macrophages and lymphocytes. Macrophages are usually more numerous, the prominence of lymphocytic infiltrates being low [10, 11]. When present, lymphocytes are perivascular or randomly distributed in epineurium or endoneurium. It has been suggested that T‐cells and antibodies might target a possible infectious agent (“molecular mimicry”), in the presence of the “major histocompatibility complex” (MHC) class II and co‐stimulatory molecules on the surface of an antigen‐presenting cell [13]. Debris‐filled macrophages may be of perivascular or subperineurial distribution, or randomly dispersed throughout the nerve fascicle (in association with axon) [10].

There is a prominent process of segmental demyelination and remyelination with a variety of aspects, from apparent normal myelin to completely demyelinated axons (depending of the duration and severity of the disease). Axons are usually spared, but, as described by Prineas [12], macrophages penetrate the basement membrane around nerve fibers and displace the adjacent SC, giving macrophage‐induced myelin disruption (by stripping myelin away from the body of SC) [14]. Vesicular destruction of myelin may sometimes precede macrophage invasion. These lesions are multifocal and predominate at the levels of nerve roots. They are supposed to be due to an immune response directed against a component of peripheral myelin which has not yet been identified [14, 15].

### 4.2 The classical CIDP

The classical form of CIDP is defined as a chronic polyradiculoneuropathy (developing over at least 8 weeks) manifesting as progressive, stepwise, or recurrent symmetrical proximal and distal weakness and sensory impairment in all limbs [16]. In 1975, Peter J. Dyck named it “chronic inflammatory polyradiculoneuropathy” (CIP) [17], before proposing the term “chronic inflammatory demyelinating polyradiculoneuropathy” (CIDP) [18], nowadays still used. CIDP mainly affect myelinated fibers and is characterized by segmental demyelination (corresponding to randomly distributed foci of acquired demyelination between two NoRs, along the internode) [19]. Despite primary demyelination, there is a secondary axonal loss (sometimes severe), as observed in about a quarter of the cases of Dyck et al. [17]. Inflammation is present in up to half of the cases [17, 20]. Most of the time, NB is not essential for the diagnosis of CIDP, because clinical and electrophysiological criteria are sufficient [21].

**FIGURE 3:**
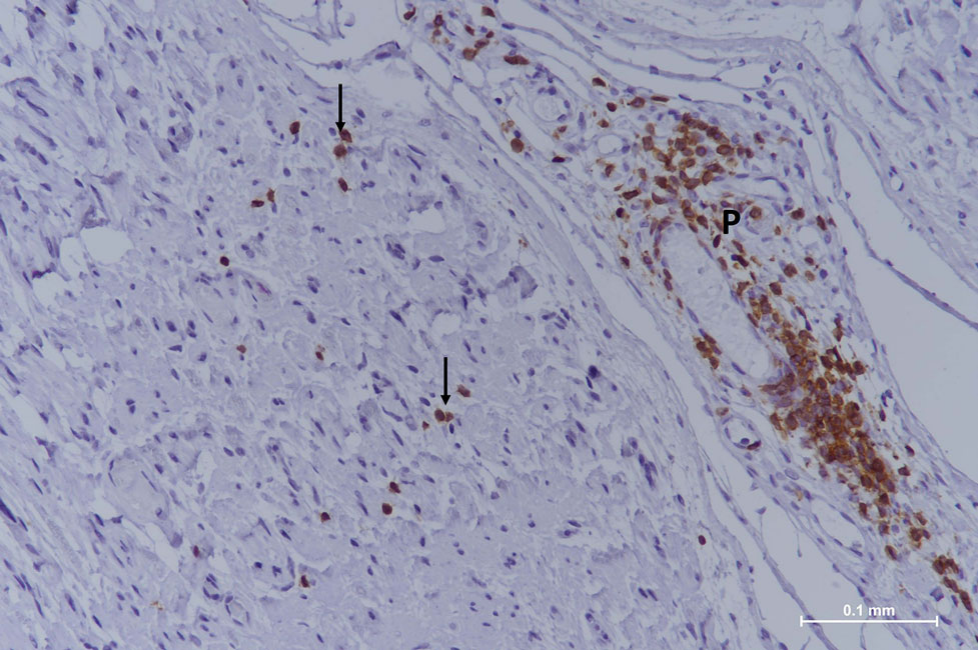
Classical CIDP. Paraffin‐embedded longitudinal section (sural nerve biopsy) with anti‐CD3 antibody staining. A vessel of the perineurium (P) is surrounded by T‐lymphocytes and a few are also scattered in the endoneurium (arrows). CIDP, chronic inflammatory demyelinating polyneuropathy.

**FIGURE 4:**
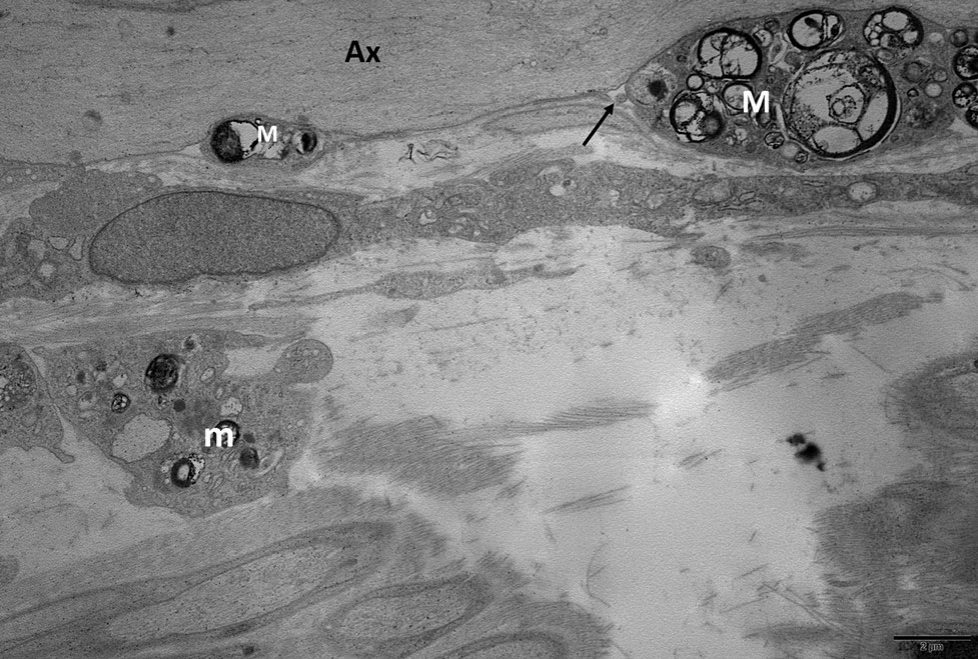
Classical CIDP. Electron micrograph, longitudinal section (sural nerve biopsy). In an internodal domain several macrophages (M) loaded with myelin debris are in contact to a large diameter axon (Ax) completely devoid of myelin; they are located between the axon and the Schwann cell basal lamina (arrow). Some other macrophages (m) are identified in the interstitial tissue. CIDP, chronic inflammatory demyelinating polyneuropathy.

In typical CIDP, the pattern of involvement is multifocal (patchy distribution of lesions across different fascicles) and resembles the one just described in AIDP [15]. By light microscopy, variable amounts of diffusely distributed mononuclear perivascular infiltrates are seen in the endoneurium and the perineurium; they are composed of macrophages and CD4+/CD8+ T‐cells, rarely B‐cells **(**Figure 3 **)** [22]. By electron microscopy, as in AIDP, the characteristic macrophage‐mediated demyelination is observed: macrophages filled with myelin debris are found within the basal membrane of SC **(**Figure 4 **)**, entering and splitting myelin sheath lamellae, and are loaded with myelin debris. Demyelinated axons may be seen on transverse toluidine blue‐stained semithin sections, although they are easier to see by electron microscopy. A significant number of large axons are surrounded by thinned myelin sheaths or completely demyelinated. Some “onion‐bulb” formations (suggestive of a chronic recurrent demyelinating‐remyelinating process) composed of proliferations of concentric flattened cytoplasmic SC processes are observed [15, 23].

In classical CIDP, it seems that nodal and paranodal regions are rarely affected [24, 25]. In fact, it is still not possible to fully explain the initial involvement by macrophages at the level of internode or node. Unidentified auto‐antibodies targeting myelin antigens probably drive this internodal myelin destruction by macrophages: several targets have been tested such as peripheral myelin protein 22 (PMP22), myelin protein zero (MPZ), E‐cadherin, myelin‐associated glycoprotein (MAG), or various gangliosides, as well as NF140/186, NF155, CNTN1 or Caspr1, but without success.

Over time, axon loss becomes more severe, so that demyelinating lesions may be difficult to identify (either electrophysiologically or pathologically); consequently, such patients are sometimes wrongly considered as having an axonal polyneuropathy and are not being treated correctly: for these reasons, it might be more appropriate to reuse the original term “CIP” rather than “CIDP” [19].

## 5 THE PATHOLOGICAL FEATURES OF NODOPARANODOPATHIES

### 5.1 Acute nodoparanodopathy

The recent concept of “nodoparanodopathy” was first applied to acute inflammatory polyradiculoneuropathy. In 1986, Feasby et al reported on five patients with “acute axonal form of GBS” they considered as a new variant of GBS: the pathological study showed sensory and motor axonal degeneration, without any evidence of segmental demyelination or inflammation [26]. Since the 1950's, many cases of “axonal GBS” similar to those of Feasby et al. have been observed worldwide, but these other cases were epidemic and seasonal (mostly affecting young subjects, often associated with enteral infection), mainly observed in China, so this entity was termed “Chinese paralytic syndrome” [7, 27]. “Axonal GBS” (including Chinese cases), are strongly associated with *Campylobacter jejuni* enteritis, IgG anti‐GM1 and anti‐GD1 antibodies in 50%–66% of patients [6, 28] This entity is nowadays known as “AMAN” or “AMSAN,” as proposed in 1993 [29].

Based on the lack of demyelinating findings and on low amplitudes of compound muscle action potential (CMAP), AMAN was first assumed to be characterized by axonal degeneration; but, in AMAN, the presence of conduction blocks (CB) and/or nerve conduction slowings (that promptly resolved without the development of excessive temporal dispersion of CMAPs, so characteristic of demyelination‐remyelination) suggests a temporary failure of conduction at the NoR possibly because of the loss of sodium voltage channels, a phenomenon called RCF to distinguish it from the classical “demyelinating CB” [4]. As observed by Griffin et al. in the motor nerve roots of a few autopsied patients, AMAN is characterized by early nodal changes consisting in the lengthening of the NoR with some distortion of the paranodal myelin [30]. In a second step, Wallerian‐like degeneration of motor fibers may be observed with no or minimal demyelination and lymphocytic infiltration [29]. So AMAN/AMSAN can be considered as nodoparanodopathies induced by anti‐ganglioside antibodies.

### 5.2 Subacute and chronic nodoparanodopathy

The term “axoglial dysjunction” was first used in 1986 by Sima et al. to describe the absence of transverse bands in the paranodes of myelinated fibers in some experimental and human diabetic polyneuropathies [31]. 10 years later, Giannini and Dyck mentioned that axonal dysjunction has been described in several pathological conditions related to de‐ and re‐myelination and presented one personal CIDP case with such lesions [32]. In 2012, Devaux et al. showed that IgG from the serum of about 30% of patients with CIDP immunolabels NoRs or paranodes of myelinated axons [5]. Then in 2013, Querol et al. identified antibodies against CNTN1 or the CNTN1/Caspr1 complex which were considered as susceptible to explain the symptoms and signs of three CIDP patients [33]. In 2016, Devaux et al. reported the clinical and serologic features of Japanese patients with CIDP and NF155 IgG4 antibodies [34]. So, these IgG4 antibodies target some specific nodal and/or paranodal proteins (NF155, NF140/186, CNTN1, or Caspr‐1) and are associated with particular clinical phenotypes of CIDP presenting pathological features of dismantling of paranodal junctions [35]. As instead the emergence of the concept of nodoparanodopathy, some cases of CIDP with IgG4 antibodies against paranodal axoglial proteins (previously considered as “atypical CIDP”) were finally included in this category [3, 36].

**FIGURE 5:**
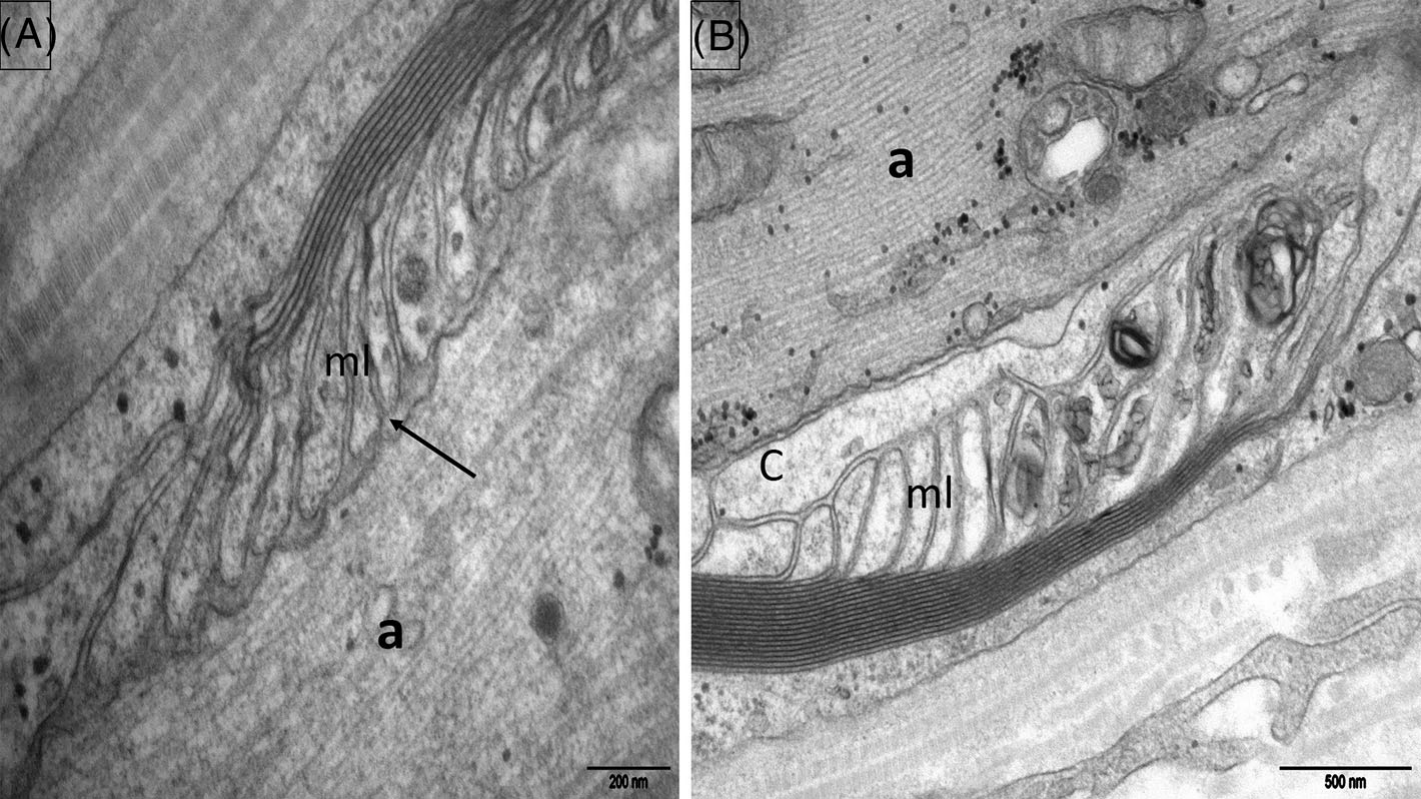
Anti‐NF155 paranodopathy. Electron micrograph, longitudinal section (sural nerve biopsy). (A): The transverse bands between the myelin loops (ml) and the axon (a) are absent (arrow). (B): A cellular process (C) is interposed between the myelin loops (ml) and the axon (a).

**FIGURE 6:**
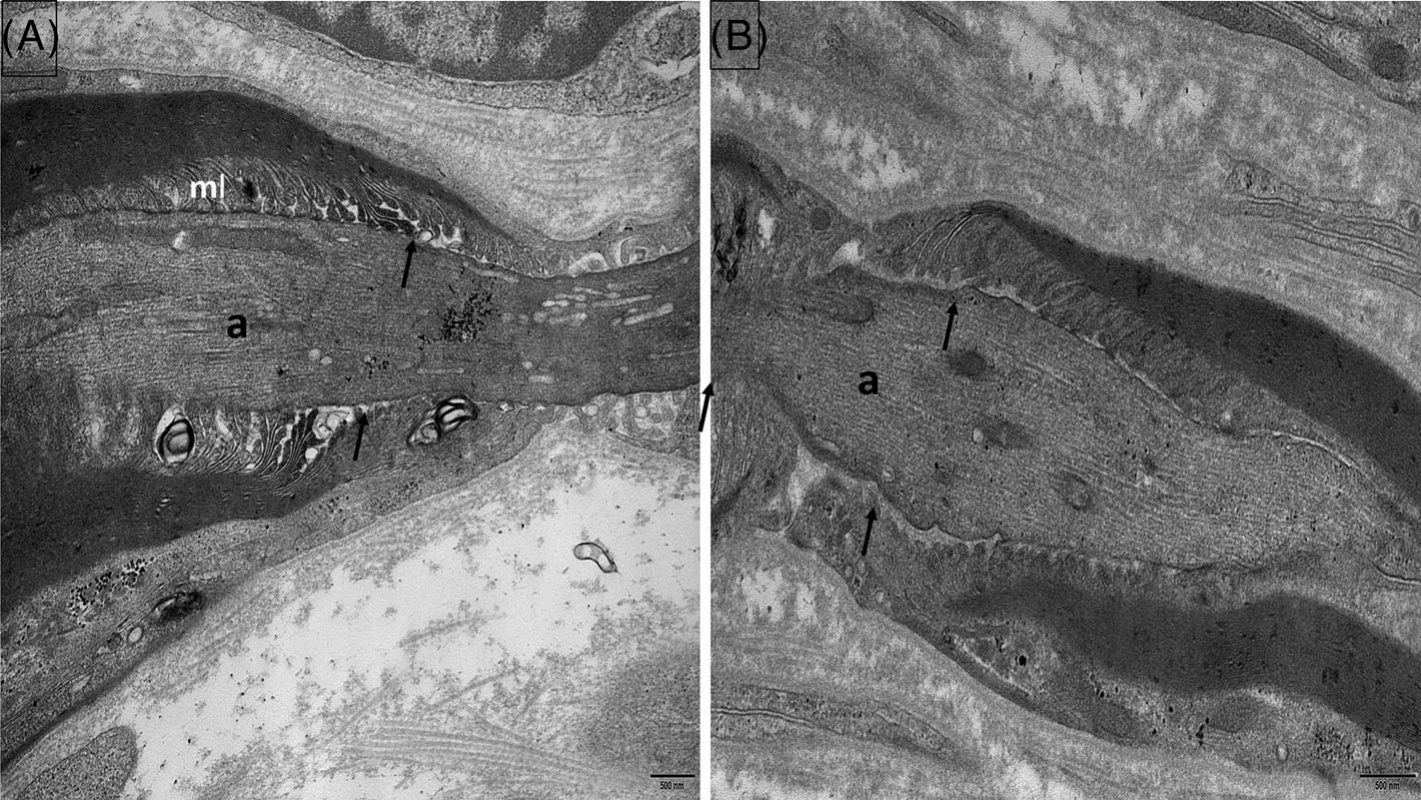
Anti‐Caspr1 (A) and anti‐pan‐neurofascin (B) paranodopathies. Electron micrographs, longitudinal sections (sural nerve biopsy). Most transverse bands are missing (arrows); myelin loops (ml) are retracted and narrow (a: axon).

Such patients present a common pathological pattern characterized by a moderate axonal loss (decrease in both large and small diameter myelinated fibers) which is not uniform between fascicles; demyelinating lesions (too thin myelin sheaths and widenings of the NoRs) and some myelin ovoids (attesting of the axonal destruction) are usually observed. [37]. Characteristic paranodal lesions are detected by EM in a significant number of paranodes, but not in all: there is a disappearance of TBs inducing a loss of attachment of the myelin loops to the axon and an irregular widening of the periaxonal space. Digitiform cellular processes from the SC cytoplasm may infiltrate this abnormally widened space and contribute to the dissociation between the axon and the paranodal loops [37] **(**Figures 5 and 6 **)**. Anti‐CNTN1 antibodies have also been shown to induce paranodal alterations identified by immunofluorescence labeling of dermal myelinated fibers of punch biopsies from CIDP patients [38].

Recently, a new entity has been identified as “anti‐pan‐neurofascin‐associated disease” because of Ig1 or IgG3 (and also IgG4) subclasses of antibodies targeting the nodal and paranodal neurofascin isoforms 140/155/186 (pan‐neurofascin): unlike IgG4, IgG1/3 can trigger Fc‐mediated effector functions including cross‐linking and complement activation, possibly mediating complement‐associated pathologies [39]. In such cases, patients usually develop a severe symptomatology (tetraplegia), monophasic (with acute onset) but potentially reversible [40, 41]. In one such case, we described the absence of microvilli with occlusion of the nodal gap by overgrowth of the elongated cytoplasm parts of two adjacent SC along the nodal axolemma; however, the paranodal regions were normal [42], but were abnormal in another personal case (Figure 6). In fact, in vitro studies demonstrated that anti‐pan‐neurofascin antibodies directly impair paranode formation and nodoparanodal architecture in mature NoR, with more severe effects than anti‐NF155 antibodies alone [41]. Furthermore, morphological alterations on myelin and sensory neurons indicate effects of both anti‐NF155 and anti‐pan‐neurofascin antibodies not only at the NoR, but in other compartments of the peripheral nerve physiologically expressing neurofascin isoforms [41].

## 6 THE PATHOLOGICAL FEATURES OF INTER‐NODOPARANODOPATHIES

“Myelin‐associated glycoprotein” is a minor constituent of myelin (<1% of all myelin proteins in both central and peripheral nervous systems), but a major component of uncompacted myelin (present in inner and outer mesaxons, Schmidt–Lanterman incisures and paranodes): it plays an important role in the axon‐myelin interactions [43]. There is a high incidence (70%) of anti‐MAG antibodies in IgM monoclonal gammopathy (MG) [44]. Anti‐MAG neuropathy is typically a “distal acquired demyelinating symmetric” (DADS) sensorimotor (mainly sensory) polyneuropathy, even if it may sometimes electrophysiologically mimick CIDP [45].

The peripheral nerve microscopical examination of patients with anti‐MAG neuropathy is characterized by some loss of myelinated fibers to different extents in different patients. The ultrastructural hallmark is the presence of “widenings of myelin lamellae” [15] which usually involves less than 5%–10% of the large, myelinated axons and are observed in both internodal and paranodal areas. Exceptionally, they may be sometimes observed in patients with IgM‐MG alone [46].

## 7 CONCLUSION

It is now well recognized that auto‐immune neuropathies are characterized by a variety of clinical phenotypes and pathological nerve lesions, which have to be considered specifically for each case to implement suitable immunotherapy. Further studies are needed to better understand the heterogeneous pathogenesis of auto‐immune neuropathies.

## AUTHOR CONTRIBUTIONS

Jean‐Michel Vallat and Stéphane Mathis both draft and revise the manuscript. Jean‐Michel Vallat did the pathological analysis.

## CONFLICT OF INTEREST STATEMENT

The authors declare no conflict of interest.

## Data Availability

Data sharing is not applicable to this article as no new data were created or analyzed in this study.
